# The Role of Stone Materials, Environmental Factors, and Management Practices in Vascular Plant-Induced Deterioration: Case Studies from Pompeii, Herculaneum, Paestum, and Velia Archaeological Parks (Italy)

**DOI:** 10.3390/plants14040514

**Published:** 2025-02-08

**Authors:** Alessia Cozzolino, Giuliano Bonanomi, Riccardo Motti

**Affiliations:** 1 Department of Agricultural Sciences, University of Naples Federico II, Via Università, 100, 80055 Portici, Italy; alessia.cozzolino2@unina.it (A.C.); giuliano.bonanomi@unina.it (G.B.); 2 Task Force on Microbiome Studies, University of Naples Federico II, 80138 Naples, Italy

**Keywords:** biodeterioration, higher plants, cultural heritage, archaeological sites, hazard index

## Abstract

The biodeterioration process involves the alteration of stone monuments by living organisms, such as bacteria, algae, fungi, lichens, mosses, ferns, and vascular plants, combined with abiotic factors, resulting in physical and chemical damage to historic buildings. This study aims to investigate the role of the vascular plants affecting four archaeological parks in Campania—Pompeii, Herculaneum, Paestum, and Velia—by analyzing correlations with building materials, exposure, and conservation status. To represent species associations and their coverage percentages at each site, transects of one square meter were employed. The hazard index (HI) was applied to evaluate the impact of the identified biodeteriogens. A total of 117 species were detected across 198 samples collected from the four study sites, with 59 taxa recorded in Pompeii, 56 in Paestum, 41 in Velia, and 36 in Herculaneum. Specifically, Pompeii hosts a predominance of cosmopolitan species (35%) and widely distributed taxa (15%) due to elevated anthropogenic disturbance. Conversely, mediterranean species dominate in Paestum (62%) and Herculaneum (52%), reflecting more stable ecological conditions. Substrate type significantly influences the hazard index, whereas exposure was found to have minimal impact on both the average coverage and the measured hazard index. Future work will focus on developing site-specific conservation strategies that consider substrate properties, vegetation impact, and anthropogenic disturbances to effectively mitigate the biodeterioration risks posed by vascular flora in Italian monumental sites.

## 1. Introduction

The conservation of archaeological sites is a significant challenge, primarily due to the impact of both abiotic factors and biological organisms. These elements contribute to the gradual degradation of materials and structures, often accelerating the weathering processes. Consequently, preserving archaeological sites requires a multifaceted approach that addresses both environmental factors and the biological agents responsible for biodeterioration [1,2]. In stone monuments, the ability of plants to colonize is influenced by the rock’s chemical properties, the microclimatic conditions related to exposure and inclination, and prior interactions with other biodeteriorative factors. This susceptibility to biological colonization is known as bioreceptivity [3]. Bioreceptivity is determined by the physical and chemical characteristics of the material and its history of chemical and biological weathering [4]. For example, porous rocks have higher bioreceptivity because they absorb and retain more water [5]. Therefore, building materials vary in their bioreceptivity and susceptibility to biological degradation. Environmental factors like moisture, wind, and temperature significantly impact the colonization and growth of living organisms on stone surfaces of monuments and artworks. As these environmental conditions persist, they promote the establishment of living communities, leading to the biodeterioration of the stone surfaces [6]. In recent decades, many researchers have focused on the role of living organisms, particularly vascular plants, in the colonization and deterioration of stone-built cultural heritage [7,8,9,10,11,12,13,14]. The growth of higher plants on walls typically requires crevices, fractures, and interstices [15]. The colonization of stone monuments by higher plants also depends on local factors, including human disturbances, microclimatic conditions like temperature and humidity, and interactions with other plants [16]. Moreover, the technology used in the construction of the walls, as well as the surrounding vegetation, affects plant growth and the type of vegetation able to colonize such anthropic habitats [15,17]. Plant growth on walls is a dynamic process where weeds can gradually alter the physical conditions of their substrate, often leading to the disintegration of building materials. Deteriogenic species are so defined because they induce various types of damage to the substrate they colonize, leading to cracks, structural collapses, and material detachment, primarily through biophysical and biochemical processes [18]. Biochemical deterioration occurs through assimilatory processes, where organisms extract nutrients from the stone surface, and dissimilatory processes, where organisms produce metabolites that chemically interact with the stone [19,20,21]. Natural vegetation in monumental areas is often a concern because plants can damage monuments with their roots, create an impression of neglect, obstruct visitor access, or obscure the monuments themselves [22]. Studying plant communities in archaeological areas, including their distribution and bioindicator values, is crucial for assessing the risk of damage to man-made structures. Knowing the spatial and numerical distribution of plant species within these areas can significantly improve site management and conservation efforts [23,24]. At archaeological sites, the aim is not to completely eradicate higher plants but to manage vegetation to an acceptable level. This task can be particularly challenging in Mediterranean environments. In this regard, it is crucial to carefully study the flora growing on walls, assessing their invasiveness, aggressiveness, and potential to cause permanent damage to the artifacts. According to data from UNESCO (the United Nations Educational, Scientific, and Cultural Organization), Italy is home to two-thirds of the world’s cultural heritage. The Campania region in southwestern Italy is one of the Italian regions with the most archaeological sites, covering the period from the establishment of Greek colonies to the fall of the Western Roman Empire [25]. The region has been celebrated for its fertile soil, strategic location, numerous natural harbors, and extensive communication networks [26]. Among the numerous archaeological sites in the Campania region, the Archaeological Park of Pompeii, the Archaeological Park of Paestum and Velia and the Archaeological Park of Herculaneum stand out for their historical importance and extension and are also among the most visited archaeological sites in the world. Given these considerations, the aims of this study were to analyze the impact of vascular deteriogenic flora and evaluate how stone construction materials, local environmental conditions, and routine management practices affect the growth and distribution of these plants in the four archaeological parks, as well as to assess the risk of structural biodeterioration.

## 2. Results and Discussion

### 2.1. Floristic Surveys

**Figure 1:**
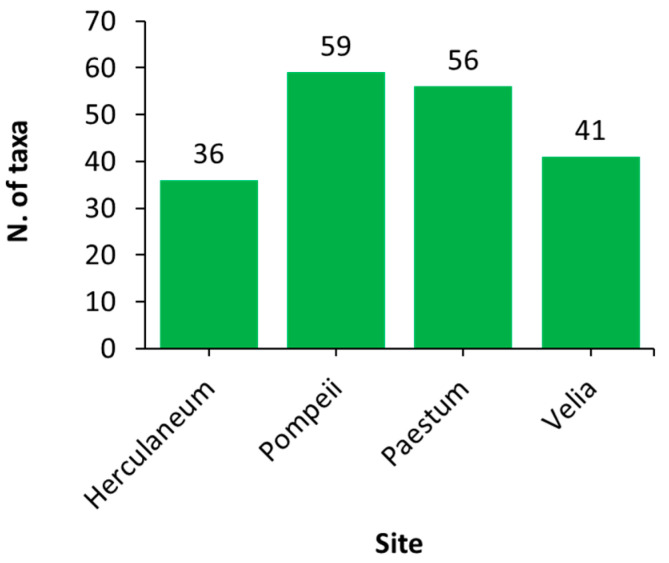
Number of taxa in the four study sites.

A total of 117 plant species were recorded (Supplementary Material Table S1), belonging to 46 families. The most species-rich families were Asteraceae (20 taxa), followed by Poaceae (17 taxa) and Fabaceae (9 taxa). *Parietaria judaica* L. (54 occurrences) was the most commonly reported species in the 198 samples, followed by *Campanula erinus* L. (27 occurrences), *Reichardia picroides* L. (27), and *Sonchus tenerrimus* L. (26). In Figure 1, the number of taxa at four archaeological sites is shown. Pompeii has the highest number of taxa, with 59, indicating a rich diversity of deteriogenic plant species at this location; Paestum follows closely with 56 taxa, also displaying considerable biodiversity; Velia has a moderate count of 41 taxa, while Ercolano records the lowest number of taxa, with only 36, which may suggest a less diverse flora compared to the other sites. The differences in the number of species observed across the sites can be attributed to several factors, including the intensity and type of maintenance practices employed. A significant factor to consider is the environmental conditions of the different locations. Pompeii and Paestum are situated near urban areas but are also largely surrounded by natural and/or cultivated environments. This proximity allows them to benefit from a potential seed bank of species from semi-natural and anthropogenic habitats, which may contribute to their higher species’ richness. In contrast, Velia, although located within an agricultural or natural context, experiences greater aridity, which can limit plant diversity. These drier conditions may restrict the number of species that can thrive in this area. Lastly, Herculaneum is entirely encompassed by an urban environment, which typically offers less favorable conditions for biodiversity.

**Figure 2:**
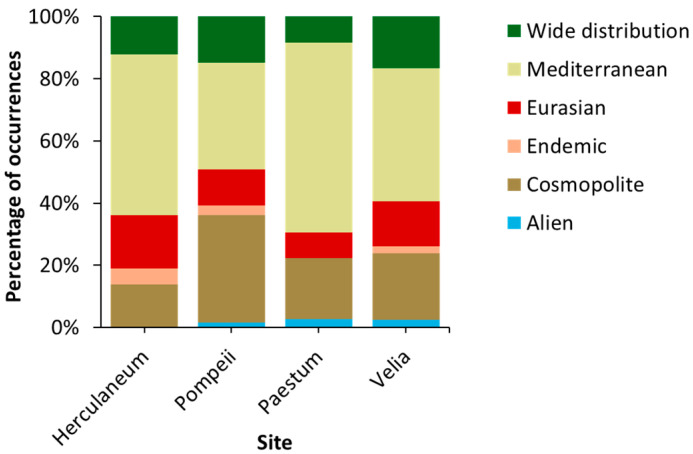
The chorological spectrum at the four sites.

**Figure 3:**
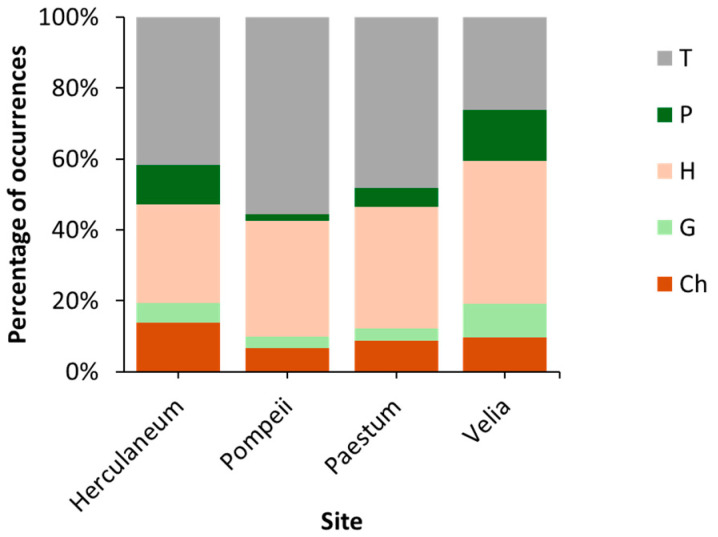
Life forms at the four sites (T = therophytes; P = phanerophytes; H = hemicryptophytes; G = geophytes; Ch = chamaephytes).

The normal chorological spectrum (Figure 2) reveals the dominance of Mediterranean species (46%), followed by cosmopolitan species (23%), and Eurasian and widely distributed species (13%). Mediterranean species are most abundant in Herculaneum (52%) and Paestum (62%), while cosmopolitan species dominate in Pompeii (35%) and Velia (22%). Eurasian species are more prominent in Herculaneum (17%) and Velia (14%), whereas widely distributed species are most represented in Pompeii (15%) and Velia (17%). The life form spectrum (Figure 3) also confirms the Mediterranean characteristic, with a predominance of therophytes (45%), followed by hemicryptophytes (34%) and chamaephytes (9%) across all study sites. Therophytes are most abundant in Pompeii (56%) and Paestum (48%), hemicryptophytes are more prevalent in Velia (40%) and Paestum (34%), while chamaephytes are most common in Herculaneum (14%) and Velia (10%).

**Figure 4:**
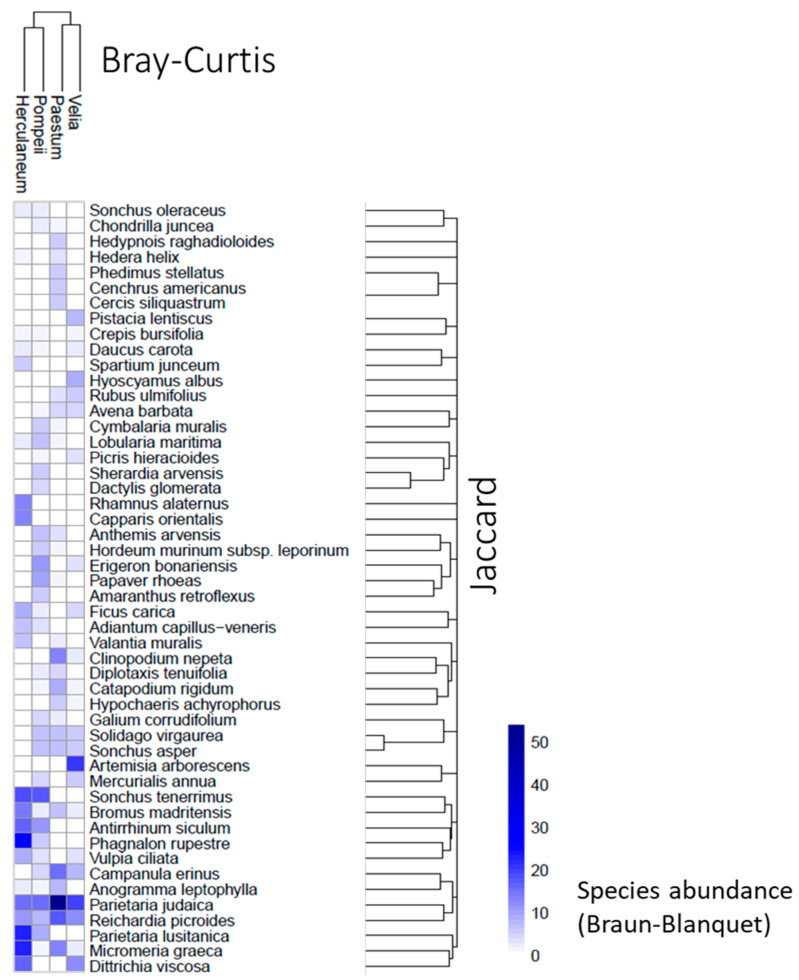
Heatmap of relative abundance of different plant species in plant communities at the four different sites. Hierarchical clustering of sites is based on Bray–Curtis, while plant species are ordered according to association index.

The chorological and life forms highlight distinct ecological dynamics across the four sites, shaped by varying degrees of anthropogenic disturbance, substrate characteristics, and habitat stability. Herculaneum is dominated by Mediterranean species, reflecting a strong regional biogeographical influence despite its urbanized context. The prominent presence of chamaephytes suggests adaptations to volcanic substrates and water-stress conditions. In contrast, Pompeii exhibits a dominance of cosmopolitan and widely distributed species, indicative of high levels of anthropogenic disturbance and a dynamic environment. The prevalence of therophytes at Pompeii highlights both the site’s instability, resulting from frequently disturbed conditions that favor annual species, and the effectiveness of maintenance and cleaning activities. Paestum, on the other hand, is dominated by Mediterranean species, with a balanced representation of therophytes and hemicryptophytes. This combination suggests relatively stable ecological conditions compared to Pompeii and Herculaneum, but still with some degree of disturbance that supports annual strategies. The balance between stability and disturbance at Paestum highlights the transitional nature of its habitats, with both stable niches and dynamic areas. Velia stands out for its high floristic and functional heterogeneity. The coexistence of Mediterranean, cosmopolitan, and widely distributed species indicates the availability of diverse microhabitats and relatively lower levels of disturbance compared to the other sites. The balanced representation of therophytes, hemicryptophytes, and chamaephytes further illustrates this ecological diversity, with the site supporting a wide range of plant strategies and habitat types. The Bray–Curtis dendrogram (Figure 4) shows that Paestum and Velia have the most similar floristic compositions among the four sites, suggesting that they may share similar ecological conditions or a common floristic history. These two sites are located in the Cilento area of the Campania region, which has a richer and more complex floristic composition than the Vesuvius area, where the other two sites are located. Additionally, the key species based on average coverage are shown: *Phagnalon rupestre* (L.) DC., *Parietaria lusitanica* L., and *Micromeria graeca* (L.) Benth. ex Rchb. for the Herculaneum site; *Parietaria judaica* L., *Reichardia picroides* L., and *Campanula erinus* L. for Paestum; *Sonchus tenerrimus* L., *Parietaria judaica*, and *Antirrhinum siculum* Mill. for Pompeii; and *Artemisia arborescens* L., *Parietaria judaica*, and *Dittrichia viscosa* (L.) Greuter for Velia.

### 2.2. Substrate Analysis

**Figure 5:**
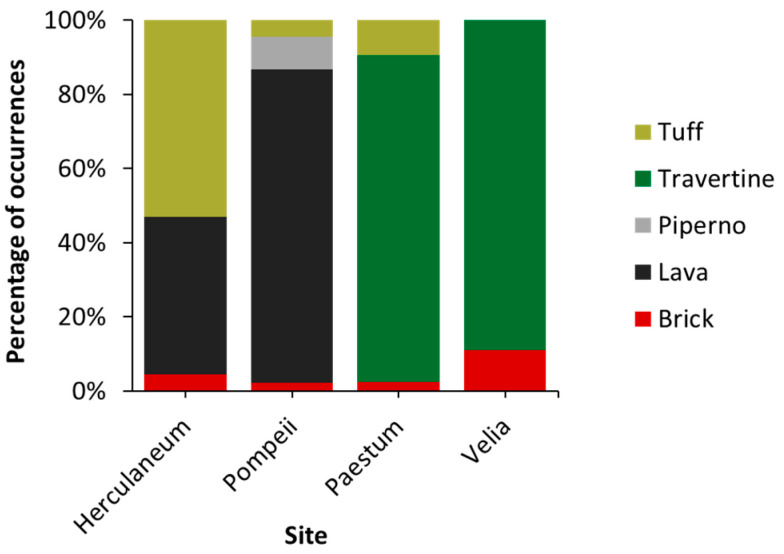
Type of substrates at the four study sites.

**Figure 6:**
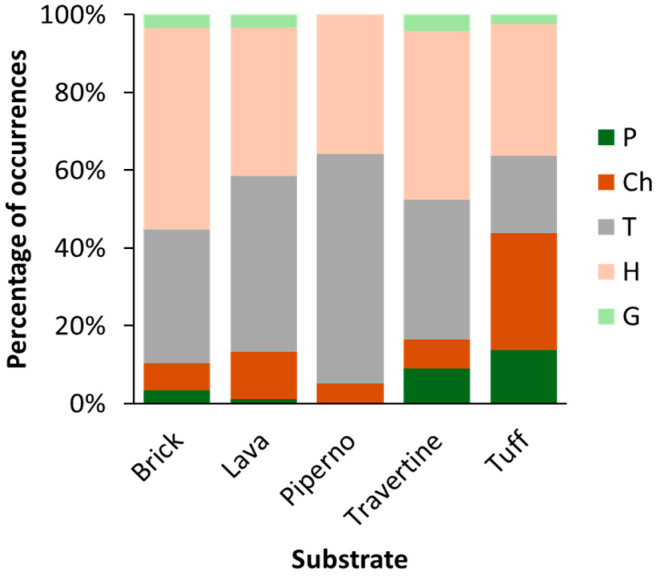
Life forms in the different substrates at the four study sites (P = phanerophytes; Ch = chamaephytes; T = therophytes; H = hemicryptophytes; G = geophytes).

Five substrate types across the four study sites were identified: brick, lava, piperno, travertine, and tuff (Figure 5). The most prevalent substrates are travertine (36%), lava (35%), and tuff (22%). Travertine is found exclusively in Paestum and Velia, where it accounts for 88% and 89% of the substrates, respectively. Lava is present only in Pompeii (84%) and Herculaneum (43%), while tuff is most abundant in Herculaneum (53%) and Paestum (10%). An analysis of the distribution of life forms across substrates (Figure 6) revealed a general dominance of therophytes and hemicryptophytes on all substrates, except tuff, where hemicryptophytes and chamaephytes are the most prominent. Phanerophytes are primarily associated with tuff and travertine. The presence of chamaephytes and phanerophytes in tuff can be attributed to the material’s high porosity and low bulk density depending on the high pumice content [27].

### 2.3. Effects on Exposures and Substrates Parameters

**Figure 7:**
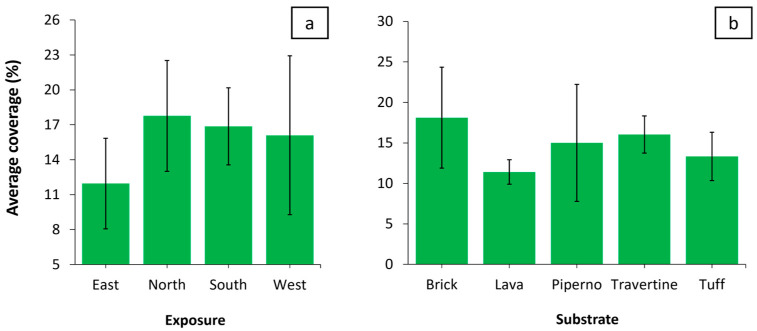
Vegetation coverage for exposure (**a**) and substrate (**b**) parameters. Error bars indicate the standard error of the mean (SEM) for the measured variables.

Figure 7a illustrates the distribution of average vegetation coverage across the four exposures. While no statistically significant differences were observed among the exposures, the results can still be interpreted. The average coverage values for south (S), west (W), and north (N) are relatively similar, whereas east (E) exhibits a visibly lower average coverage. East-facing areas receive sunlight during the cooler morning hours, which can accelerate the evaporation of dew or surface moisture. Although this early light may benefit certain plants, exposure to cold morning winds can hinder growth, particularly in environments with limited protection or insufficient nutrients. In contrast, south-facing exposures receive the most direct and intense sunlight throughout the day. This orientation creates warm and bright conditions that favor plants adapted to high light levels and heat. However, these conditions can also pose challenges for drought-sensitive species, as water stress becomes a critical factor. West-facing areas are exposed to strong sunlight in the afternoon, coinciding with peak daily temperatures. This creates a warm microclimate that can promote the growth of species requiring heat for optimal development, although it may also increase water demands for less heat-tolerant plants. On the other hand, north-facing exposures remain cooler and more humid due to their limited direct sunlight. These conditions are particularly suitable for species that thrive in shaded or moisture-rich environments, as reduced solar intensity minimizes water loss and supports a more stable microclimate. Figure 7b shows the distribution of vegetation coverage across different substrate types. Although no statistically significant differences were detected among the substrates, brick and travertine exhibited the highest average coverage, while lava displayed the lowest. The higher vegetation coverage observed on brick and travertine substrates can be explained by the physical characteristics of these materials. Due to their high porosity and the presence of matrix fissures, historical bricks often display low apparent specific densities (1.5–1.8 kg/m^3^), high water absorption rates (13–30%), and moderate compressive strengths (5–20 MPa). Similarly, travertine is characterized by notable porosity and a degree of friability [28,29].

### 2.4. Hazard Index

The hazard index is an effective tool for assessing the level of risk posed by deteriogenic species to the areas they colonize. Its value is determined by considering both the life form of the plant, which includes its size and life cycle traits, and its cover within a given area. This integrated approach allows for a more precise assessment of the potential impact of different species on the built heritage, making the hazard index a valuable metric for assessing biodeterioration risks [6].

**Figure 8:**
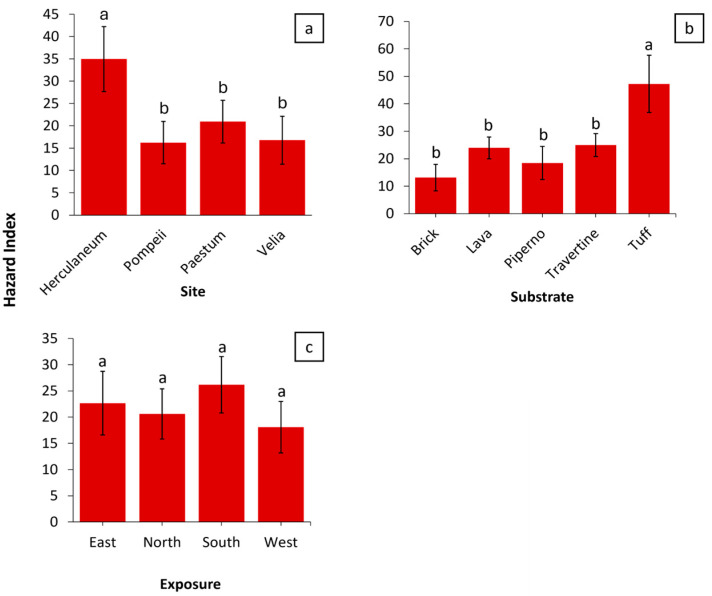
The average hazard index (HI) across the different sites (**a**), substrates (**b**), and exposures (**c**). Error bars represent the standard error of the mean (SEM). Bars sharing the same letter are not significantly different according to the Duncan test (*p* < 0.05).

Figure 8a illustrates that Herculaneum exhibits the highest hazard index (HI) among the analyzed sites. In Pompeii, the low HI value can be attributed to the dominant presence of lava and piperno substrates (Figure 5), both of which are renowned for their compactness and structural integrity. The low open porosity of Vesuvian lavas results in minimal differences between bulk and apparent density, consequently reducing total water absorption and capillary absorption coefficients [30]. Regarding piperno, previous studies have shown that erosion and vegetation have limited influence on its weathering processes [31]. In this way, these two substrates limit the growth of species with a vigorous root system. In Paestum Park and Velia, travertine is the predominant material, with moderate amounts of tuff also present in Paestum. Travertine is characterized by a complex porous structure, including vugular and fenestral macropores, which enhance water retention and make this substrate highly bioreceptive [32]. Despite this, both Paestum and Velia display low HI values. This discrepancy may be attributed to the influence of maintenance activities, which are likely more frequent on these sites. Tuff, on the other hand, is particularly susceptible to weathering due to its high zeolite content and the inherent heterogeneity of the rock, factors that contribute to water-induced weakening [33,34]. The abundance of tuff at Herculaneum, combined with less frequent maintenance compared to the other sites, likely explains its elevated hazard index.

The hazard index is certainly closely linked to maintenance activities, as archaeological sites implement periodic cleaning practices, including the manual removal of herbaceous species, along with cutting and the subsequent application of selective herbicides to control and eradicate woody species, particularly on vertical surfaces.

The hazard index (HI) values for the analyzed substrates (Figure 8b) reflect a strong relationship between vegetation cover, the physical properties of the materials, and the distribution of life forms (Figure 6). Brick and piperno exhibit low hazard index (HI) values, 13.1 and 18.5, respectively. For brick, this can be attributed to its significant resilience to environmental stress factors [35], while for piperno, the low HI value is likely due to its intrinsic resistance and compact matrix [36]. In both cases, these substrates are predominantly colonized by plant species with superficial and minimally invasive root systems, such as hemicryptophytes (H) and therophytes (T), which together account for approximately 80% of the vegetation present on these materials. Conversely, more porous substrates like travertine and tuff display moderate to high HI values (24.9 and 47.2, respectively), driven by their vulnerable structure and the presence of more aggressive life forms, such as chamaephytes (Ch) and phanerophytes (P), which have deeper root systems capable of penetrating the material and promoting degradation. Lava represents an intermediate case, with a moderate HI (23.9) despite low vegetation cover (11.4%), attributed to the presence of pioneer species with root systems adapted to exploit microcracks. The hazard index (HI) values differed without significant differences between the four exposures, but still explain the role of climatic conditions in influencing substrate degradation and vegetation colonization (Figure 8c). The southern (S) exposure shows the highest HI, likely due to increased solar radiation and greater temperature fluctuations, which enhance substrate weathering and promote the establishment of aggressive plant species, such as phanerophytes and chamaephytes. The eastern (E) exposure has a moderately high HI, which may be explained by moderate humidity levels and reduced direct sunlight during parts of the day, creating conditions that favor plant species with adaptable root systems. Conversely, the north (N) and west (W) exhibit low hazard index (HI) values. In the north, this is attributed to limited solar exposure and cooler temperatures, which inhibit the growth of aggressive plant species with deep root systems. Nonetheless, the cooler and shaded conditions may still support the presence of less impactful plant species, thereby reducing the overall risk of degradation. In the west, persistent humidity combined with higher afternoon sunlight creates unsuitable conditions for the colonization of vigorous species, further contributing to the lower HI values.

## 3. Materials and Methods

### 3.1. Study Sites

**Figure 9:**
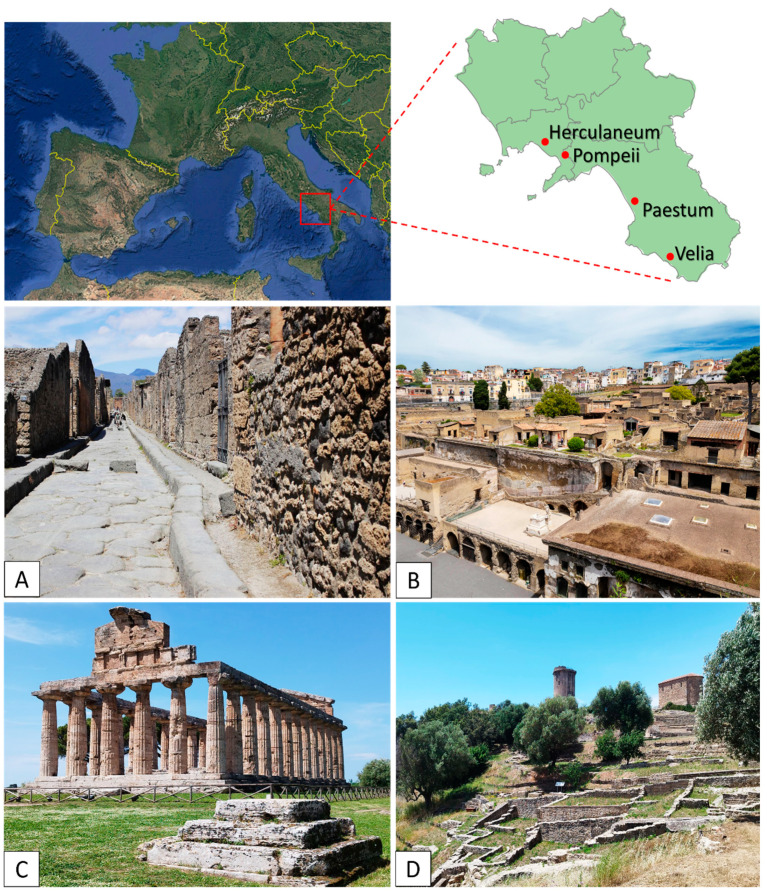
Study sites: (**A**) Pompeii; (**B**) Herculaneum; (**C**) Paestum; (**D**) Velia. Pictures by Alessia Cozzolino.

The study was conducted at four archaeological sites located in the Campania region, Italy: the Archaeological Parks of Pompeii, Herculaneum, Paestum, and Velia (Figure 9).

#### 3.1.1. The Archaeological Park of Pompeii

The Archaeological Park of Pompeii (APP) was listed as a World Heritage Site on 6 December 1997, together with Herculaneum and Torre Annunziata [37]. According to data from the official website [38], the site recorded nearly 4 million visitors in 2023, ranking among the 10 most visited archaeological sites worldwide [39]. The APP is located in the municipality of Pompei in the Metropolitan City of Naples, on the slopes of Mount Vesuvius. When Vesuvius erupted on 24 August AD 79, it engulfed the two flourishing Roman towns of Pompei and Herculaneum, as well as the many wealthy villas in the area. It was discovered at the end of the 16th century, but excavation began only in 1748, thanks to Charles of Bourbon, the King of Naples. Systematic excavations continued throughout the 19th century, leading up to the most recent efforts in excavation, restoration, and enhancement of the city and its exceptional architectural, sculptural, painting, and mosaic heritage. The archaeological area extends nowadays over approximately 66 hectares, of which about 45 hectares have been excavated. It includes villas, houses, public buildings, entertainment buildings (a theatre and an amphitheater) as well as a necropolis.

#### 3.1.2. The Archaeological Park of Herculaneum

The Archaeological Park of Herculaneum (APH) is in the municipality of Ercolano, in the Metropolitan City of Naples. As well as Pompeii, Herculaneum was buried under volcanic ash and pumice in the eruption of Mount Vesuvius in 79 AD. According to the traditional account, the city was rediscovered by chance in 1709 during the drilling of a well. However, remnants of the city had already been uncovered during earlier earthworks. In 1710, while constructing a private villa, workers discovered a theatre that was one of the most decorated public buildings in the ancient city of Herculaneum. From that moment on, under the aegis of Charles of Bourbon, excavations continued, gradually uncovering the ruins of the ancient, buried city [40]. The total area enclosed by the walls of the ancient city is thought to have been around 20 hectares, of which around 4.5 hectares are now visible. It includes houses, villas, public buildings, thermae, and a gymnasium. Since 2001, the Herculaneum Conservation Project, a public–private partnership that includes the Packard Humanities Institute, a philanthropic foundation, and a team of public heritage officials, has been active. In 2023, the APH registered approximately 600,000 visitors, according to data from the official website [41].

#### 3.1.3. The Archaeological Park of Paestum and Velia

The Archaeological Park of Paestum and Velia (APPV) comprises two archaeological sites: one in Paestum, a district of the municipality of Capaccio, and the other near the modern village of Novi Velia, close to the municipality of Ascea. Both sites are in the province of Salerno and are evidence of coastal human settlements during the Magna Graecia period along the Cilento coast. The most notable features of the Paestum site today are the three large temples, with massive colonnades in the archaic version of the Greek Doric order, dating to around 550-450 BC [42]. An amphitheatre, a *Comitium*, and a Roman forum also reveal the life of the city, with its customs and organization. The total area enclosed by the walls of the ancient city amounted to 25 hectares. The site of Velia covers an area of about 100 hectares and includes a medieval tower built on the ruins of the ancient Greek theatre, two gates, buildings, thermae, agora, and an amphitheatre. Regarding visitor frequency, the (APPV) recorded nearly 500,000 visitors, according to the official website [43].

### 3.2. Data Collection and Analysis

Field samples were collected between March and September 2024. In total, we conducted 198 vegetation surveys (Table 1). At each site, surfaces were randomly chosen for the surveys. For each selected surface a central 1 × 1-m sampling unit was analyzed to represent different floristic types in terms of plant cover and floristic diversity.

The following data were recorded for each sampling unit: site name, position (UTM coordinates), substrate, and exposure. Plant cover was recorded on the Braun–Blanquet abundance–dominance scale [44] and then transformed into percentage values as follows: 5 = 88%; 4 = 63%; 3 = 38%; 2 = 15%; 1 = 5%; + = 1%. To assess the hazard posed by deteriogenic species to buildings, a hazard index (HI) [45,46] was assigned for each taxon. This index is a numerical scale ranging from 1 (minimal hazard) to 10 (high hazard) and is based on three parameters: plant life form, invasiveness and vigor, and root system characteristics. For the analysis, the product of the hazard index and the mean coverage value for each species was used. Plant specimens were identified in the field, with uncertain cases later examined at the Laboratory of Applied Ecology, Department of Agricultural Sciences at Portici following Flora d’Italia [47,48] and Flora Europaea [49,50]. Plant nomenclature followed the World Flora Online [51], while angiosperm families were classified according to the APG IV system [52]. Abbreviations of authors were standardized following [53]. Plant life forms were classified following [54] and were largely verified through field observations. The chorotype was assigned according to [47,55,56]. To assess differences in plant community composition, we performed a cluster analysis of the study sites using the Bray–Curtis similarity index. Furthermore, plant species within the communities were ranked according to their association index, emphasizing those species that contributed most significantly to community differentiation. The analysis of the association index was restricted to the 50 species with the highest cumulative abundance at each site. A one-way analysis of variance (ANOVA) was performed to evaluate the effects of different parameters, using SPSS statistical software 29.0, with significance assessed at *p* < 0.05. Post-hoc comparisons were conducted using Duncan’s test.

## 4. Conclusions

The data collected in this study provide significant insights into the spontaneous vascular flora colonizing Italian monumental sites. The archaeological sites analyzed, renowned for their substantial cultural and historical importance, underscore the urgent need to address the colonization of plant species that can accelerate deterioration processes. Key factors influencing these processes include the type of building material (substrate), site exposure, and the characteristics of the present vegetation. These factors, alongside the surrounding environmental conditions, are further shaped by the degree of anthropogenic disturbance at each site, determining the potential risk of structural damage. Moreover, while vegetation cover plays a critical role, the hazard index is strongly modulated by the interplay between the physical properties of the substrates and the impact of dominant life forms. This finding highlights the importance of monitoring biological colonization to deepen our understanding of degradation processes and to develop effective, site-specific mitigation strategies. Considering substrate properties, the degree of anthropogenic disturbance, and the surrounding ecological context allows for identifying the most at-risk sites and prioritizing maintenance interventions accordingly. Our recommendation is to continue routine maintenance activities while paying closer attention to the areas most at risk. This involves optimizing both the frequency and type of interventions, ensuring a targeted strategy. In particular, maintenance efforts should distinguish between species with high hazard index values, considering their interaction with the substrate type, and thus the vulnerability of the colonized surface, and the surrounding environmental context. This adaptive management approach would improve the conservation of archaeological structures by mitigating biodeterioration more effectively.

## Figures and Tables

**Table 1:** The number of samples carried out at each study site.

Site	Municipality	N. of Surveys
Pompeii	Pompei	46
Herculaneum	Ercolano	69
Paestum	Capaccio Paestum	46
Velia	Ascea	37

## Data Availability

Data are available upon request.

## Supplementary Materials

The following supporting information can be downloaded at: https://www.mdpi.com/article/10.3390/plants14040514/s1. Supplementary Table S1: Plant species recorded at Archaeological Parks of Pompei, Herculaneum, Paestum and Velia.
