# Supplementary material for: The Role of Stone Materials, Environmental Factors, and Management Practices in Vascular Plant-Induced Deterioration: Case Studies from Pompeii, Herculaneum, Paestum, and Velia Archaeological Parks (Italy)
Source: Plants (Basel). 2025 Feb 8;14(4):514. doi: 10.3390/plants14040514 (PMC11859654; doi:10.3390/plants14040514)
Supplement: Supplementary file 1 [file plants-14-00514-s001.zip › plants-3444597-supplementary.pdf]

Table S1. Plant species recorded at Archaeological Parks of Pompei, Herculaneum, Paestum and Velia

**Species**	**Family**	**Chorology**	**Plant life form**	**HI**
Acanthus mollis L.	Acanthaceae	Doubtful alien [Mediterranean]	H scap	4
Adiantum capillusveneris L.	Pteridaceae	Palaeotropical	G rh	3
Ajuga chamaepitys (L.) Schreb.	Lamiaceae	EuriMedit.	T er	2
Amaranthus retroflexus L.	Amaranthaceae	Cosmop.	T er	3
Anogramma leptophylla (L.) Link	Hemionitidaceae	Subtrop.	T er	1
Anthemis arvensis L. subsp. *incrassata* (Loisel.) Nyman	Asteraceae	StenoMedit.	T er	2
Antirrhinum siculum Mill.	Plantaginaceae	Endemic	H scap	6
Arenaria serpyllifolia L. subsp. *serpyllifolia*	Caryophyllaceae	Cosmop.	T er	1
Artemisia arborescens (Vaill.) L.	Asteraceae	SMedit.	NP	8
Asparagus acutifolius L.	Asparagaceae	StenoMedit.	G rh	6
Asplenium ceterach L. subsp. *ceterach*	Aspleniaceae	Eurasiat.	H ros	1
Avena barbata Pott ex Link	Poaceae	Medit.Turan	T er	2
Blackstonia perfoliata (L.) Huds. subsp. *intermedia* (Ten.) Zeltner	Gentianaceae	EuriMedit.	T er	1
Bromus diandrus Roth	Poaceae	EuriMedit.	T er	3
Bromus hordeaceus L. subsp. *hordeaceus*	Poaceae	StenoMedit.	T er	2
Bromus madritensis L.	Poaceae	EuriMedit.	T er	4
Calendula arvensis (Vaill.) L.	Asteraceae	StenoMedit.	H bienn	4
Campanula erinus L.	Campanulaceae	StenoMedit.	T er	2
Capparis orientalis Veill.	Capparaceae	Eurasiat.	NP	8

Catapodium rigidum (L.) C.E.Hubb. subsp. *rigidum*	Poaceae	EuriMedit.	T er	1
Celtis australis L. subsp. *australis*	Cannabaceae	EuriMedit.	P scap	10
Cenchrus americanus (L.) Morrone	Poaceae	Subcosmop.	T er	2
Cercis siliquastrum L. subsp. *siliquastrum*	Fabaceae	Eurasiat.	P scap	10
Chondrilla juncea L.	Asteraceae	EuriMedit.Sudsib.	H scap	4
Clinopodium vulgare L. subsp. *orientale* Bothmer	Lamiaceae	Circumbor.	H scap	5
Crepis bursifolia L.	Asteraceae	Endem.	H scap	2
Cymbalaria muralis P. Gaertner, B. Meyer et Scherb. subsp. *muralis*	Plantaginaceae	Subcosmop.	Ch rept	4
Cynodon dactylon (L.) Pers.	Poaceae	Subcosmop.	H rept	4
Cynosurus echinatus L.	Poaceae	EuriMedit.	T er	2
Dactylis glomerata L.	Poaceae	Paleotemp.	H scap	4
Dactylis hispanica Roth	Poaceae	StenoMedit.	H scap	4
Daucus carota L.	Apiaceae	Subcosmop.	T er	4
Digitaria sanguinalis (L.) Scop.	Poaceae	Cosmop.	T er	4
Diplotaxis tenuifolia (L.) DC.	Brassicacee	EuriMedit.Subatl.	H scap	4
Dittrichia viscosa (L.) W. Greuter	Asteraceae	EuriMedit.	Ch suff	8
Echium vulgare L.	Boraginaceae	Europ.	H bien	4
Equisetum ramosissimum Desf.	Equisetaceae	Circumbor.	G rh	1
Erigeron bonariensis L.	Asteraceae	Avv. (America trop.)	T er	4
Euphorbia peplus L.	Euphorbiaceae	Cosmop.	T er	1
Festuca myuros L.	Poaceae	Subcosmop.	T er	2

Ficus carica L.	Moraceae	Medit.Turan.	P m	10
Foeniculum vulgare Miller	Apiaceae	SMedit.(Steno)	H scap	5
Fumaria capreolata L.	Papaveraceae	EuriMedit.	T scd	1
Galium album Miller	Rubiaceae	EuropeoWAsiatico	H scap	1
Galium aparine L.	Rubiaceae	Eurasiat.	T scd	1
Galium corrudifolium Vill.	Rubiaceae	StenoMedit.	H scap	2
Geranium molle L.	Geraniaceae	Subcosmop.	T er	2
Geranium robertianum L.	Geraniaceae	Subcosmop.	T er	2
Geranium rotundifolium L.	Geraniaceae	Paleotemp.	T er	2
Glebionis coronaria (L.) Cass. ex Spach	Asteraceae	StenoMedit.	T er	2
Hedera helix L.	Araliaceae	EuriMedit.Subatl.	P l(Ch suff)	7
Hedypnois rhagadioloides (L.) Willd.	Asteraceae	StenoMedit.	T er	2
Hordeum murinum L subsp. *leporinum* (Link) Arcangeli	Poaceae	EuriMedit.	T er	2
Hyosciamus albus L.	Solanaceae	EuriMedit.	Ch suff	5
Hypericum perforatum L.	Hypericaceae	Subcosmop.	H scap	6
Hypochaeris achyrophorus L.	Asteraceae	StenoMedit.	T er	2
Hypochaeris radicata L.	Asteraceae	EuropeoCaucas.	H ros	2
Lactuca muralis (L.) Gaertn.	Asteraceae	EuropeoCaucas.	H scap	2
Lamium purpureum L.	Lamiaceae	Eurasiat.	T er	1
Linum strictum L.	Linaceae	StenoMedit.	T er	1
Lobularia maritima (L.) Desv.	Brassicaceae	StenoMedit.	Ch suff	4

Lolium perenne L.	Poaceae	Circumbor.	H caesp	3
Lysimachia arvensis (L.) U.Manns & Anderb.	Primulaceae	Cosmop.	T rept	2
Malva sylvestris L.	Malvaceae	Subcosmop.	H scap	5
Medicago minima (L.) L.	Fabaceae	EuriMedit. Centroasiat.	T er(rept)	1
Mercurialis annua L.	Euphorbiaceae	Paleotemp.	T er	2
Micromeria graeca (L.) Bentham ex Reichenb.	Lamiaceae	StenoMedit.	Ch suff	6
Myosotis arvensis Hill	Boraginaceae	EuropeoWAsiat.	T er	2
Olea europaea L.	Oleaceae	StenoMedit.	P m	10
Oxalis corniculata L.	Oxalidaceae	Cosmop.	H rept	2
Oxalis debilis Kunth	Oxalidaceae	Alien	G bulb	2
Papaver rhoeas L.	Papaveraceae	Cosmop.	T er	1
Parietaria judaica L.	Urticaceae	SEMedit.(Euri)	H scap	5
Parietaria lusitanica L.	Urticaceae	StenoMedit.	T er	1
Phagnalon rupestre (L.) DC.	Asteraceae	W e SMedit.	Ch suff	7
Phedimus stellatus	Crassulaceae	StenoMedit.	T succ	2
Picris hieracioides L.	Asteraceae	Eurosib.	H scap	2
Pistacia lentiscus L.	Anacardiaceae	StenoMedit.	P n(m)	10
Plantago lanceolata L.	Plantaginaceae	Cosmop.	H ros	3
Portulaca oleracea L.	Portulacaceae	Subcosmop.	T er	3
Raphanus raphanistrum L.	Brassicaceae	Circumbor.	T er	4
Reichardia picroides (L.) Roth	Asteraceae	StenoMedit.	H scap	6

Reseda alba L.	Resedaceae	StenoMedit.	H scap	6
Rhamnus alaternus L.	Rhamnaceae	StenoMedit.	P n(m)	10
Rubus ulmifolius Schott	Rosaceae	EuriMedit.	P n	10
Rumex crispus L. subsp. *crispus*	Polygonaceae	Subcosmop.	H scap	4
Salvia verbenaca L.	Lamiaceae	Medit.Atl.	H scap	4
Sanguisorba minor Scop.	Rosaceae	Subcosmop.	H scap	4
Sedum dasyphyllum L.	Crassulaceae	EuriMedit.	Ch succ	4
Senecio vulgaris L.	Poaceae	Cosmop.	T er	1
Setaria verticillata (L.) P. Beauv.	Poaceae	Subcosmop.	T er	2
Sherardia arvensis L.	Rubiaceae	Subcosmop.	T rept	1
Silene gallica L.	Caryophyllaceae	Subcosmop.	T er	1
Smilax aspera L.	Smilacaceae	Paleosubtrop.	P l	8
Solanum nigrum L.	Solanaceae	Cosmop.	T er	4
Solidago virgaurea L.	Asteraceae	Circumbor.	H scap	4
Sonchus asper (L.) Hill	Asteraceae	Subcosmop.	H scap	2
Sonchus oleraceus L.	Asteraceae	Subcosmop.	H scap	2
Sonchus tenerrimus L.	Asteraceae	StenoMedit.	H scap	2
Sorghum halepense (L.) Pers.	Asteraceae	Subcosmop.	G rh	6
Spartium junceum L.	Fabaceae	EuriMedit.	P n	10
Symphytum bulbosum C. Schimper	Boraginaceae	SEEurop.	G rh	2
Trachelium coeruleum L.	Campanulaceae	WMedit.(Steno)	H scap	4

Trifolium campestre Schreber	Fabaceae	(W)Paleotemp.	T er	2
Trifolium nigrescens Viv.	Fabaceae	EuriMedit.	T er	2
Trifolium repens L.	Fabaceae	Subcosmop.	H scap	5
Trigonella esculenta Willd.	Fabaceae	NMedit.(Steno)	T er	3
Umbilicus horizontalis (Guss.) DC.	Crassulaceae	StenoMedit.	G b	3
Urtica membranacea Poiret	Urticaceae	SMedit.(Euri)	T er	1
Valantia muralis L.	Rubiaceae	StenoMedit.	T rept	1
Valeriana locusta L.	Caprifoliaceae	EuriMedit.	T er	1
Verbascum sinuatum L.	Scrophulariaceae	EuriMedit.	H scap	4
Verbena officinalis L.	Verbenaceae	Cosmop.	H scap	2
Veronica polita Fries	Plantaginaceae	Subcosmop.	T rept	1
Vicia pseudocracca Bertol.	Fabaceae	StenoMedit.	T scd	2
Vicia sativa L.	Fabaceae	Subcosmop.	T scd	2
Vulpia ciliata Dumort.	Poaceae	EuriMedit.	T er	2
